# HOG-Independent Osmoprotection by Erythritol in Yeast *Yarrowia lipolytica*

**DOI:** 10.3390/genes11121424

**Published:** 2020-11-27

**Authors:** Dorota A. Rzechonek, Mateusz Szczepańczyk, Guokun Wang, Irina Borodina, Aleksandra M. Mirończuk

**Affiliations:** 1 Department of Biotechnology and Food Microbiology, Wrocław University of Environmental and Life Sciences, 50-375 Wrocław, Poland; dorota.rzechonek@upwr.edu.pl (D.A.R.); mateusz.szczepanczyk@upwr.edu.pl (M.S.); 2 The Novo Nordisk Foundation Center for Biosustainability, Technical University of Denmark, 2800 Kongens Lyngby, Denmark; guowan@biosustain.dtu.dk (G.W); irbo@biosustain.dtu.dk (I.B.)

**Keywords:** *Yarrowia lipolytica*, erythritol, hyperosmotic stress response, osmolytes

## Abstract

Erythritol is a polyol produced by *Yarrowia lipolytica* under hyperosmotic stress. In this study, the osmo-sensitive strain *Y. lipolytica yl-hog1Δ* was subjected to stress, triggered by a high concentration of carbon sources. The strain thrived on 0.75 M erythritol medium, while the same concentrations of glucose and glycerol proved to be lethal. The addition of 0.1 M erythritol to the medium containing 0.75 M glucose or glycerol allowed the growth of *yl-hog1Δ*. Supplementation with other potential osmolytes such as mannitol or L-proline did not have a similar effect. To examine whether the osmoprotective effect might be related to erythritol accumulation, we deleted two genes involved in erythritol utilization, the transcription factor Euf1 and the enzyme erythritol dehydrogenase Eyd1. The strain *eyd1Δ yl hog1Δ*, which lacked the erythritol utilization enzyme, reacted to the erythritol supplementation significantly better than *yl-hog1Δ*. On the other hand, the strain *euf1Δ yl-hog1Δ* became insensitive to supplementation, and the addition of erythritol could no longer improve the growth of this strain in hyperosmotic conditions. This indicates that Euf1 regulates additional, still unknown genes involved in erythritol metabolism.

## 1. Introduction

Erythritol is a four-carbon polyol, which is used as a sugar substitute. It gained popularity as a sweetener because of interesting properties such as a very low caloric value, little impact on insulin release [1], and indications that its usage might lower the prevalence of caries-related mouth bacteria [2]. Unlike many synthetic sweeteners, it can be found naturally in food such as fruits or honey [3]. Yet, the role of this compound in metabolism is still unclear. Erythritol is often described as a zero-calorie sweetener, due to its fast absorption in the small intestine and subsequent excretion of most of the intake with the urine [4]. Initially, it was believed that erythritol was not metabolized in the human body. However, the most recent studies revealed that a small fraction of the intake could be processed to erythronate [5]. Moreover, erythritol could be synthesized from glucose in the blood and mammalian tissues [5,6], and in some cases elevated erythritol levels in blood might be a marker of upcoming changes in the health condition [5,7]. Thus, there is a need for better understanding of the role of this polyol in metabolism.

As erythritol is becoming a more widely available food additive, it is necessary to ensure that long-term usage of this sweetener does not have any negative impact on the human body and microflora. It is a difficult task because many aspects of erythritol metabolism remain unclear even in single-celled organisms. In this study, we focused on the role of erythritol in the yeast *Yarrowia lipolytica*. This yeast is capable of both the synthesis [8] and utilization [9] of erythritol; thus it might become a model of erythritol metabolism in eukaryotic cells. Other yeast genera that are known to produce significant amounts of erythritol are *Trigonopsis*, *Pichia*, *Moniliella*, *Pseudozyma*, *Aureobasidium*, and *Trichoderma* [3]. Some of them have interesting industrial applications or are a part of the human microbiome [10].

**Figure 1:**
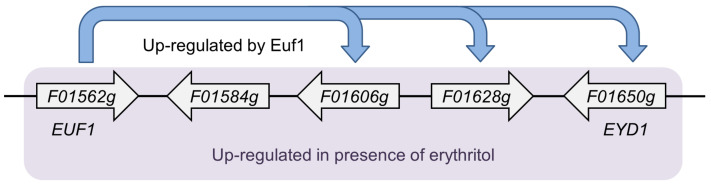
Cluster of genes involved in erythritol utilization located on chromosome F in *Y. lipolytica* CLIB122 genome [9].

*Y. lipolytica* is a dimorphic, oleaginous yeast that is being increasingly studied due to its great potential for industrial applications [11]. It produces erythritol under hyperosmotic conditions [12]. The biosynthesis of erythritol proceeds mainly via the pentose phosphate pathway (PPP) [3]. Erythrose-4-phosphate, the metabolite of PPP, is dephosphorylated by a still unknown enzyme to erythrose, which is later converted to erythritol by erythrose reductase [13]. After depletion of the original carbon source in the growth medium, the concentration of erythritol starts to decrease. There are four enzymes known to be involved in erythritol utilization: erythritol dehydrogenase Eyd1 [14], kinase Eyk1 [15], and two isomerases, Eyi1 and Eyi2 [16]. The genes encoding these proteins are regulated by the transcription factor Euf1. All of them, including *EUF1*, are arranged in a cluster in the genome called the erythritol utilization cluster (Figure 1). Euf1 is most likely not the only factor regulating erythritol utilization, since in its absence the *euf1Δ* strain may still use erythritol as a carbon source to a small extent [9].

The correlation between high osmotic pressure and erythritol production indicates that it might be an unusual stress response. After years of studies carried out on the model yeast *Saccharomyces cerevisiae* [17] and the pathogenic yeast *Candida albicans* [18], it is known that yeast responses to various environmental stresses are regulated by the network of signaling pathways. The response to osmotic stress is transmitted through the high osmolarity glycerol (HOG) pathway. The central part of the pathway is a cascade of protein kinases (MAPK cascade) and its final element is the protein kinase Hog1. Activation of the HOG pathway leads to the phosphorylation of Hog1. In this activated form, it interacts with numerous cell elements in both the cytoplasm and the nucleus, which leads to adaptation to the environment. A significant part of the response is intracellular accumulation of small molecules with a neutral charge known as osmolytes. That balances the osmotic pressure on both sides of the membrane and prevents the efflux of water. In the case of *S. cerevisiae* the main osmolyte is glycerol [19].

*Y. lipolytica* has a homologue of kinase Hog1 and its deletion leads to a high sensitivity to osmotic stress. Moreover, the strain *yl-hog1Δ* shows a few other anomalies, e.g., increased filamentous growth and higher resistance to cell wall disturbing agents [20]. Similar changes have been previously observed in *hog1Δ* deletion strains of *S. cerevisiae* [21] and *C. albicans* [22], which indicate the similarities of signaling pathways. However, the final responses are different, as the osmolyte produced by *Y. lipolytica* is erythritol rather than glycerol. The intracellular concentration of erythritol rises several-fold in hyperosmotic conditions [23], but surprisingly a significant amount of this polyol is also secreted to the environment.

In this study, we investigated the importance of erythritol in osmoprotection. We used an osmo-sensitive strain that carries the deletion of *yl-Hog1*, and combined it with modifications of genes involved in erythritol metabolism.

## 2. Materials and Methods

### 2.1. Strains and Culture Conditions

Strains used in the study were *Y. lipolytica* AJD, the *ura3Δ* derivative of *Y. lipolytica* A101, and its further derivatives. They were obtained from the Department of Biotechnology and Food Microbiology at Wrocław University of Environmental and Life Sciences, Poland. Strains of *Escherichia coli* DH5α were the carriers of plasmids containing deletion or overexpression cassettes and were used for transformation procedures. The full list of used strains and plasmids is in Table 1.

*E. coli* strains used in the transformation procedures were cultivated in LB medium. YPD medium, containing 1% (*w/v*) yeast extract (Merck, Darmstadt, Germany), 1% (*w/v*) peptone (Merck, Darmstadt, Germany), and 2% (*w/v*) glucose (Chempur, Piekary Śląskie, Poland), was used for yeast inoculum preparation. YNB (Sigma-Aldrich, Darmstadt, Germany) agar plates with 2% (*w/v*) glucose were used for standard yeast transformations. YPD agar plates with Hygromycin B (800 µL/100 mL) were used for yeast transformations to restore auxotrophy. YPD agar plates with 2% (*w/v*) NaCl were used to screen for osmo-sensitive strains. YNB agar plates with varying concentrations of carbon sources were used in stress sensitivity tests. The carbon sources were: glucose, glycerol (Wratislavia-Biodiesel, Wroclaw, Poland), or erythritol (Młyn Oliwski, Gdańsk, Poland). The plates for stress sensitivity tests were also supplemented with various concentrations of additions such as erythritol, mannitol (Sigma-Aldrich, Darmstadt, Germany), and L-proline (Sigma-Aldrich, Darmstadt, Germany). Glycerol, glucose, and erythritol used in the media were sterilized by filtration and added to other compounds, just before the preparation of the agar plates. All yeast strains were cultivated at 30 °C.

Shake-flask experiments were performed in 0.3 L flasks with baffles containing 0.05 L of medium kept on a rotary shaker at 240 rpm. Media used for these experiments were: YNB medium with 100 g/L glycerol and optional supplementation of 10 g/L erythritol. 3 g L^−1^ CaCO_3_ was also added in order to prevent a drop in pH during culture.

### 2.2. Analytical Methods

Osmolality of some media was measured on the osmometer OS 3000 Marcel, Poland. The samples were diluted 10-fold before measurement. The concentrations of polyols and organic acids were determined by HPLC using a HyperRez Carbohydrate H+ Column (Thermo Scientific, Waltham, MA, USA) coupled to a UV (*λ* = 210 nm) (Dionex, Sunnyvale, CA, USA) and a refractive index detector (Shodex, Ogimachi, Japan). 0.25% trifluoroacetic acid was used as a mobile phase solvent. The samples were diluted 10-fold before the measurement. Data were analyzed with the Chromeleon program. Samples for biomass measurements (5 mL) were collected from shake-flask cultures and harvested by filtration on 0.45 µm pore size membranes. The biomass was determined gravimetrically after drying in a drier at 105 °C.

### 2.3. Deletion and Overexpression Cassettes

Plasmids containing deletion or overexpression cassettes prepared in previous studies were purified from *E. coli* strains on a Plasmid Mini kit (A&A Biotechnology, Gdynia, Poland). The *yl-hog1* deletion cassette was carried on plasmid pQE80-ptHog1+Ura [20], the *euf1* deletion cassette was on plasmid pUC-ura-ΔF01562 [9], and the *EUF1* overexpression cassette was on plasmid pAD-F01562 [9].

The deletion cassette for the gene *eyd1* was prepared as follows. The gene YALI0F01650 promoter region was amplified by PCR with Phusion high-fidelity DNA polymerase (Thermo Scientific, Darmstadt, Germany) and primers p1650-F-PmeI (CCAGTTTAAACCTCCAAGAGCTGCCACTGTAG) and p1650-R-NotI (TTAGCGGCCGCGGAAACTGTTGTCAGTATTTG). The obtained PCR product was digested with the enzymes MssI and NotI and inserted into corresponding sites of the plasmid pUC-Ura [9] with the ligation enzyme T4 DNA Ligase (Thermo Scientific, Darmstadt, Germany), resulting in the vector pUC-Ura-pF01650. The proper integration was tested by PCR reaction with primers p1650-col-F (GGAATAAGGCTGGTCATGATGG) and p1650-col-R (CAGAGTACGGCTTCTCAATCG). Transformation of *E. coli* strains was performed using standard chemical protocols [27].

The YALI0F01650 terminator fragment was amplified with primers t1650-F-ApaI (ATTGGGCCCTCATCCCGACTGACAGCTAAC) and t1650-R-HindIII (GATAAGCTTTCAGC GGGAAGCTGGAGAG). The PCR product was digested with the enzymes ApaI and HindIII and inserted into the plasmid pUC-URA-pF01650. The final plasmid pUC-Ura-ΔF01650 was tested for proper integration with PCR reaction with the primers t1650-col-F (AATAATCTCGGGACAGCAATG) and t1650-col-R (TTGTGTAGGGCTTACTTGATG).

### 2.4. Yeast Transformation

Transformation of *Y. lipolytica* was performed according to the lithium acetate method [28]. The strain AJD with auxotrophy for uracil was transformed with one of the plasmids containing an overexpression or deletion cassette and transformants were plated out on selective media without uracil. They were tested for proper integration by gDNA extraction and PCR reaction. The uracil auxotrophy was restored by transformation with the replicative plasmid pUB4-Cre1 [25] with the Cre-lox recombinase system. The transformants were plated out on YPD medium with hygromycin B. After restoring the auxotrophy and loss of the replicative plasmid, strains were transformed with the plasmid pQE80-ptHog1+Ura [20]. The proper integration was confirmed with PCR reaction on isolated gDNA and increased sensitivity to growth on YPD plates with 2% NaCl.

The strain AJD was also transformed with the plasmid pAD. The plasmid was used for preparation of overexpression cassettes. However, in this case the cassette was empty and the plasmid was only allowed to integrate the gene *URA3* into the rDNA sequence. The resulting AJDU strain was used as a control, instead of the original A101, because removal of *URA3* from the original locus caused some differences in growth rate between A101 and AJD strains.

### 2.5. Stress Sensitivity Tests

*Y. lipolytica* strains were grown to the exponential phase (OD600 = 0.6) in liquid YPD medium. The dilutions 1, 10^−1^, 10^−2^, and 10^−3^ were spotted on YNB agar plates, with 0.5 M, 0.75 M, or 1 M concentration of one of the carbon sources—glucose, glycerol, or erythritol. YNB media with glucose and glycerol were also supplemented with erythritol (0.01–0.1 M), mannitol (0.1 M), or L-proline. Photos of the plates were taken after 24, 48, and 72 h of cultivation. The tests were performed in duplicate.

## 3. Results

Previous research on yl-Hog1 in *Y. lipolytica* has shown that the deletion strain *yl-hog1Δ* is very sensitive to osmotic stress induced by addition of NaCl to the growth media [20]. In this study, we focused on high concentrations of carbon sources as a stressing factor. The initially chosen carbon sources were glucose, glycerol, and erythritol. The osmotic pressure of the medium is related to the molar concentration of its compounds. Thus, we compared media with the same molar concentration of the carbon source. It should be noted that glycerol, glucose, and erythritol have different molar masses, and the concentrations given in g L^−1^ are significantly different. There was also a possibility that the chosen carbon sources might interact differently with other compounds of the media, or there might be losses during the sterilization process. To prevent such factors influencing further research, the osmolality values of YNB medium with 0.5 M carbon sources were measured. The result was 780 ± 34 mOsm kg^−1^ H_2_O for medium with glycerol, 733 ± 41 mOsm kg^−1^ H_2_O for glucose and 743 ± 49 mOsm kg^−1^ H_2_O for erythritol. 

**Figure 2:**
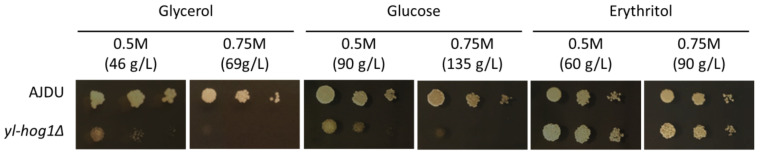
Growth of *Y. lipolytica* AJDU and *yl-hog1Δ* after 48 h of incubation on YNB agar plates with different carbon sources.

First, it has to be determined what concentration of carbon sources might inhibit the growth of strain *yl-hog1Δ*. The sensitivity of *Y. lipolytica* AJDU (control) and AJD *yl-hog1Δ* was tested on YNB plates containing 0.5 M or 0.75 M of one of the substrates: glucose, glycerol, or erythritol (Figure 2). The cultures were carried out for at least 72 h. Photos presented in the figures were taken in the 48th hour, as the changes between tested strains were the most clearly visible.

Changes in the concentration had little effect on the growth of the control AJDU strain, but the strain *yl-hog1Δ* was very vulnerable to a rise in substrate concentration. The impact of media containing glucose and glycerol was similar: 0.5 M was enough to severely diminish the growth compared to the control strain and the growth was completely inhibited in 0.75 M. However, the results for erythritol-containing media were surprisingly different. On 0.5 M erythritol, the strain *yl-hog1Δ* grew better than the control. Colonies on 0.75 M were slightly smaller, but the growth was still not weaker than the control. To ensure that the weaker growth of strain *yl-hog1Δ* on glycerol and glucose was caused by their too high concentrations, we additionally prepared plates with 0.3 M carbon sources (Supplementary Materials, Figure S1). With these lower concentrations, the development of strain *yl-hog1Δ* on glycerol and glucose was significantly improved.

**Figure 3:**
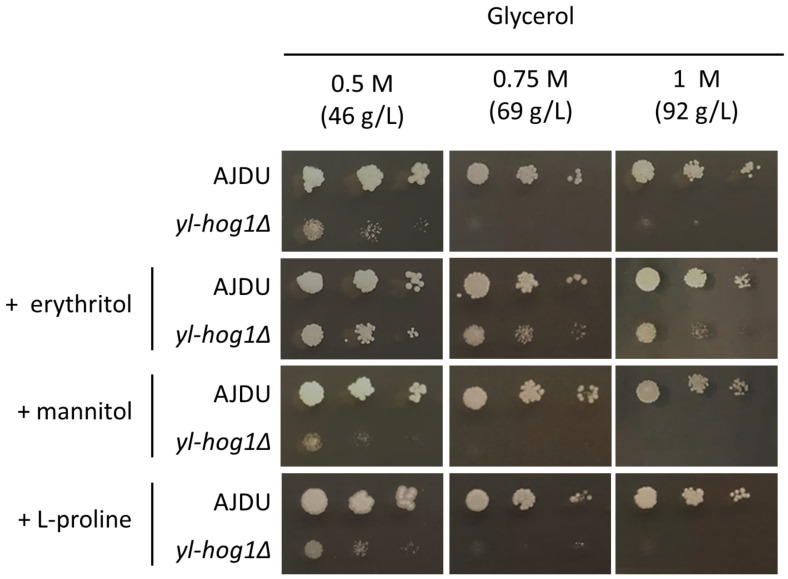
Growth of *Y. lipolytica* AJDU and *yl-hog1Δ* after 48 h of incubation on YNB agar plates with glycerol as the main carbon source. The additives tested for osmoprotective properties were 0.1 M erythritol, 0.1 M mannitol, and 0.02 M proline.

The next question was whether erythritol might improve the growth of *yl-hog1Δ* when mixed with another substrate that generates hyperosmotic stress. To test this possibility, YNB agar plates with 0.5 M, 0.75 M, or 1 M glycerol and 0.1 M erythritol supplementation were prepared. As observed in a previous experiment, 0.75 M concentration of glycerol could completely inhibit the development of *yl-hog1Δ.* However, when the erythritol was also present in the medium, the deletion strain could endure not only 0.75 M glycerol, but also 1 M (Figure 3). The addition of erythritol did not completely overcome the osmotic stress though, as the strain *yl-hog1Δ* was still significantly weaker than the control.

There was a possibility that the beneficial influence on *yl-hog1Δ* might be achieved by supplementing the hyperosmotic media with other compounds with osmoprotective properties. The most common osmolyte for yeast is glycerol, but in the case of *yl-hog1Δ* it was used as a stressing factor. From the group of other potential osmoprotectors we decided to use mannitol and L-proline. Mannitol, like erythritol and glycerol, is a polyol. Moreover, it is one of the main byproducts of erythritol production by *Y. lipolytica* [23]. Proline is an osmoprotectant for many groups of organisms [29,30], and a several-fold rise of its intracellular concentration was also observed in *Y. lipolytica* cells subjected to osmotic stress [31]. Mannitol was added to YNB-glycerol medium to the concentration 0.1 M, similar to erythritol, but the supplementation did not improve the growth of *yl-hog1Δ*. L-proline was added in a lower concentration (0.02 M), because as an amino acid, it is a source of not only carbon, but also nitrogen. Thus, the supplementation was expected to have a large impact on yeast growth. Colonies of the control strain were indeed bigger, but *yl-hog1Δ* was still not able to grow in higher concentrations of glycerol. To ensure that the difference between erythritol and proline supplementation was not the result of a lower concentration of proline, YNB-glycerol plates with erythritol supplementation from 0.01 to 0.05 M were prepared. The concentration of 0.02 M erythritol could still improve the growth of *yl-hog1Δ*, although to a lesser extent than 0.1 M concentrations.

Next, we attempted to learn more about the mechanism of the protection. Osmolytes are known to be accumulated inside cells to balance the high osmotic pressure of the environment. To test the importance of erythritol accumulation, we targeted two proteins associated with its utilization: the transcription factor Euf1 and the enzyme Eyd1.

**Figure 4:**
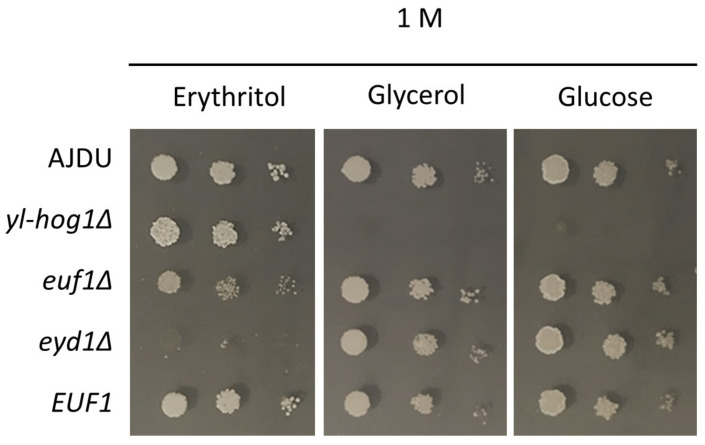
Growth of *Y. lipolytica* AJDU (control), *yl-hog1Δ*, *euf1Δ*, *eyd1Δ*, and *EUF1* after 48 h of incubation on YNB agar plates with 1 M glycerol, glucose, and erythritol as the main carbon source.

The two deletion strains *euf1Δ* and *eyd1Δ* were created on an AJD background. *EUF1* was also overexpressed in the strain *Y. lipolytica EUF1*. The properties of the new transformants were tested on YNB plates with 1 M erythritol, glucose or glycerol as a sole carbon source (Figure 4). Both Euf1 and Eyd1 are known to be important for erythritol utilization. However, cultivation on erythritol as a single carbon source revealed the difference in the phenotype of deletion strains. The growth of *euf1Δ* was impaired, but still possible, whereas the development of *eyd1Δ* was completely inhibited. Overexpression of *EUF1* did not cause any significant changes compared to the control. To ensure that the weak growth of *euf1Δ* and *eyd1Δ* was not a result of stress caused by a high concentration of the substrate, all transformants were also grown on glucose and glycerol. In these conditions, the growth of strains *euf1Δ* and *eyd1Δ* and *EUF1* did not differ from the control.

**Figure 5:**
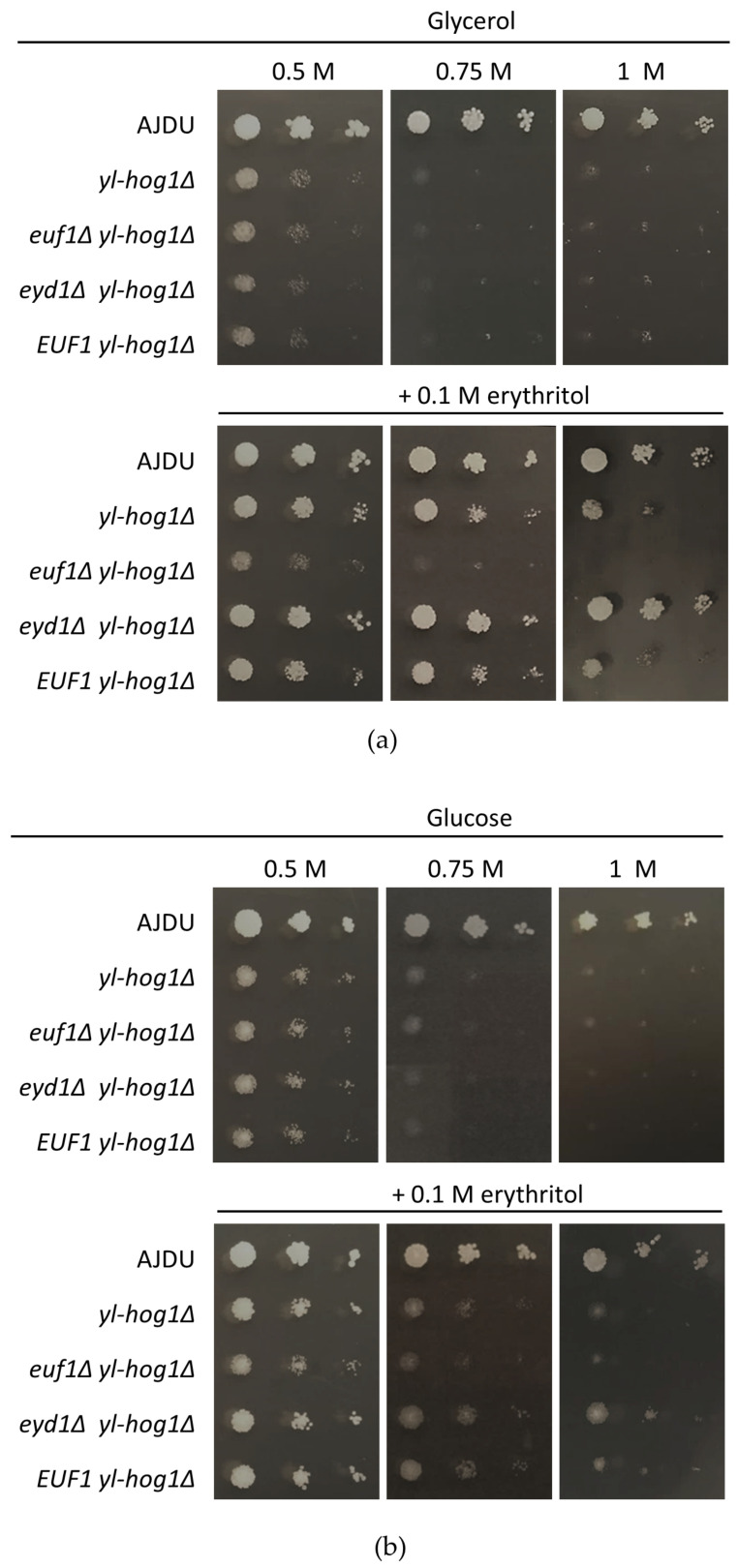
Growth of *Y. lipolytica* AJDU, *yl-hog1Δ*, *euf1Δ yl-hog1Δ*, *eyd1Δ yl-hog1Δ* and *EUF1 yl-hog1Δ* after 48 h of incubation on YNB agar plates with glycerol (**a**) or glucose (**b**) as the main carbon source and supplementation with erythritol.

After establishing that *EUF1* and *EYD1* deletions do not influence the sensitivity to osmotic stress, the deletion yl-Hog1 was added. The strains *euf1Δ yl-hog1Δ*, *eyd1Δ yl-hog1Δ*, and *EUF1 yl-hog1Δ* were all unable to grow at glucose and glycerol levels higher than 0.5 M (Figure 5). However, the addition of erythritol revealed significant differences. The strain *euf1Δ yl-hog1Δ* did not react to supplementation. Its growth was exactly the same as on plates without erythritol: weak on 0.5 M carbon sources and completely inhibited at higher concentrations. It was a striking contrast to all other tested strains. On the other hand, *eyd1Δ yl-hog1Δ* reacted to the supplementation more strongly than *yl-hog1Δ*. On the plates with glycerol as a main carbon source, the growth of *eyd1Δ yl-hog1Δ* was comparable to the control strain, regardless of the glycerol concentrations (Figure 5a). On glucose plates, *eyd1Δ yl-hog1Δ* could withstand higher glucose concentrations than other deletion strains, but there was still strong growth inhibition compared to the control strain (Figure 5b). The last created strain, *EUF1 yl-hog1Δ*, did not differ from *yl-hog1Δ* in all tested conditions.

The result that should be underlined is the significant difference between the effects of the erythritol supplementation on glucose and glycerol media. When glucose was used as the main carbon source (Figure 5b), the positive influence of erythritol was significantly weaker, although still visible.

**Figure 6:**
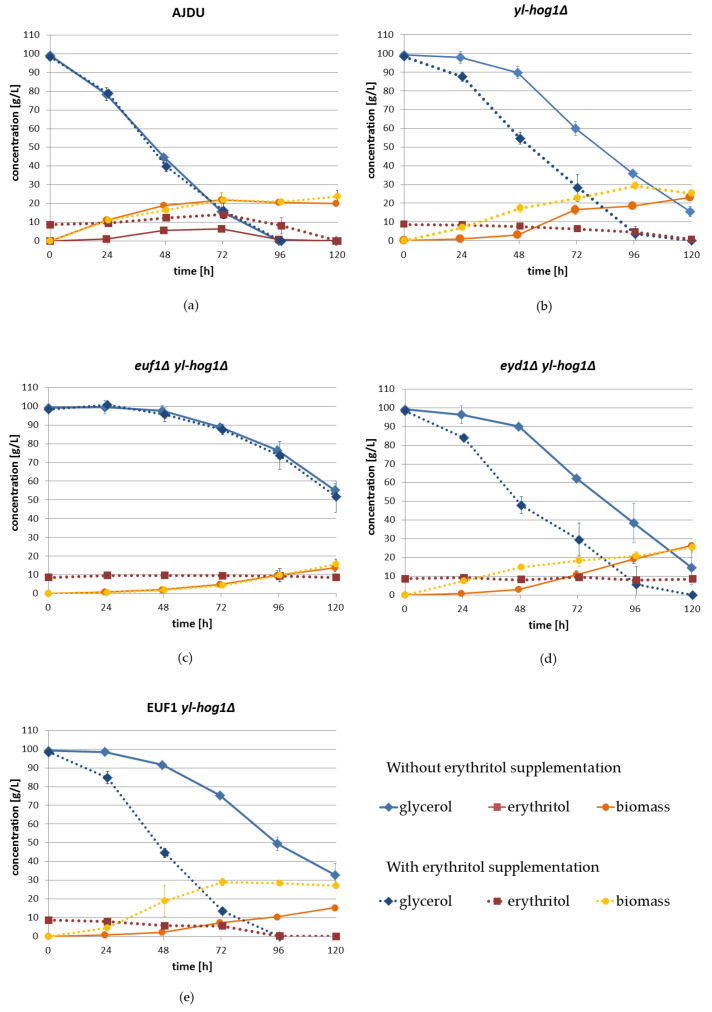
Changes of glycerol, erythritol, and biomass concentration during shake-flask cultures of *Y. lipolytica* strains: AJDU (**a**), *yl-hog1Δ* (**b**), *euf1Δ yl-hog1Δ* (**c**), *eyd1Δ yl-hog1Δ*, (**d**) and EUF1 *yl-hog1Δ* (**e**). Results from YNB glycerol (100 g L^−1^) medium without erythritol are marked with solid line, results from YNB glycerol medium with erythritol (10 g L^−1^) supplementation and are marked with dotted line. Experiment was conducted in triplicate.

To further investigate the impact of erythritol supplementation on growth, substrate utilization, and secretion, a shake-flask experiment with liquid YNB medium with 100 g L^−1^ glycerol (1.08 M) was prepared. Erythritol was supplemented in the concentration 10 g L^−1^ (0.08 M). The unit g L^−1^ was used because it is easier to monitor the carbon flux. Similar to previous experiments, the erythritol supplementation did not significantly influence the cultures of *Y. lipolytica* AJDU (Figure 6a). The addition of erythritol did not change the speed of glycerol utilization. Erythritol was produced, regardless of its initial presence in the medium. Its concentration reached 6.5 ± 0.3 g L^−1^ without supplementation and 14.1 ± 0.9 g L^−1^ with supplementation. In both cases erythritol was utilized after the depletion of glycerol.

In shake-flask cultures, all strains with deletion of yl-hog1 were found to be less sensitive to the osmotic stress generated by glycerol than in plate cultures (Figure 6). They were able to adapt to the conditions, even without erythritol supplementation, but there was still a significant delay compared to the control AJDU strain.

During the first 48 h of cultivation without supplementation, the increase in biomass and utilization of glycerol by the strain *yl-hog1Δ* were low, but significant changes appeared on the third day of cultivation (Figure 6b). By the end of the experiment (120 h), the biomass reached 23.2 ± 2.1 g L^−1^, and 15.5 ± 2.6 g L^−1^ of glycerol remained. The addition of erythritol accelerated the rate of glycerol utilization and the biomass increase. Both glycerol and erythritol were completely exhausted by the end of the experiment. Biomass reached its highest value at 96 h, 29.3 ± 2.2 g L^−1^, which was higher than that of the AJDU control.

Similar to the agar plate sensitivity tests, in shake-flask cultures the strain *euf1Δ yl-hog1Δ* was also unable to use erythritol supplementation to improve adaptation to the osmotic stress (Figure 6c). The average rates of glycerol utilization and biomass growth were very similar in cultures both with and without the addition of erythritol. Moreover, the lack of euf1 resulted in significant deterioration of the adaptability in comparison to the strain *yl-hog1Δ*. After 48 h, the utilization rate of glycerol remained very slow, and at the end of the experiment there was still 55.2 ± 3.6 g of L^−1^ of glycerol in culture without erythritol and 51.7 ± 8.4 g L^−1^ in cultures with supplementation. Biomass growth was also extremely slow compared to all other strains, and at 120 h it was 13.7 ± 2.0 g L^−1^ and 15.6 ± 2.7 g L^−1^, respectively.

The results for the strain *eyd1Δ yl-hog1Δ* were similar to those for *yl-hog1Δ*. In the absence of erythritol, at the end of the experiment, there was 14.7 ± 7.3 g L^−1^ remaining glycerol, while with supplementation, glycerol was completely depleted at 120 h (Figure 6d). The concentration of supplemented erythritol remained constant throughout the entire experiment, due to the deletion of *EYD1*. The inability to utilize erythritol could be the reason for lower final biomass (25.43 ± 2.6 g L^−1^) than for *yl-hog1Δ.*

Erythritol supplementation had the most significant impact on glycerol utilization by strain EUF1 *yl-hog1Δ* (Figure 6e). In the absence of erythritol, the adaptation was slower compared to *yl-hog1Δ* and *eyd1Δ yl-hog1Δ*. After 120 h there was 32.7 ± 6.3 g L^−1^ of glycerol and biomass concentration was 15.3 ± 1 g L^−1^. When erythritol was in the medium, both glycerol and erythritol were completely depleted in 96 h. Moreover, the biomass reached a high concentration of 29 ± 1.2 g L^−1^ in just 72 h and remained stable until the end of the experiment.

Shake-flask experiments revealed interesting differences in erythritol assimilation. The strains *yl-hog1Δ* and EUF1 *yl-hog1Δ* utilized glycerol and erythritol simultaneously. In contrast, the strain AJDU first produced erythritol, and the concentration of this polyol started to decrease only when glycerol was completely depleted. In cultures of *euf1Δ yl-hog1Δ* and *eyd1Δ yl-hog1Δ* the concentrations of erythritol remained stable. In cultures without erythritol supplementation of strains with yl-hog1 deletion, small amounts of this polyol (0.01–0.1 g L^−1^) were detected in the supernatant after 24 h of incubation until the end of the experiment.

## 4. Discussion

The correlation between erythritol production and high osmotic pressure has been observed in *Y. lipolytica* and a few other yeast species for years, and this phenomenon was used for optimization of erythritol production [3]. The research has been focused on industrial application, and thus little is known about physiological processes associated with the stress response in this yeast. *Y. lipolytica* is relatively resistant to high osmotic pressure, and some strains are isolated from the marine environment [32]. Therefore, in studies on osmotic stress, hyperosmotic conditions were provided through NaCl supplementation [20,33,34]. However, in this study we observed that the growth of the osmo-sensitive strain *Y. lipolytica yl-hog1Δ* is already significantly impaired by glycerol in the concentration 100 g L^−1^ (1.08 M) in liquid cultures (Figure 6), and on agar plates a 1 M carbon source is enough to completely inhibit the growth (Figure 2). The amount of glycerol commonly used in batch cultures is at least 100 g L^−1^; thus, in most of the research *Y. lipolytica* is subjected to conditions in which a functional HOG pathway is necessary for efficient growth.

### 4.1. Erythritol Protection Independent of HOG Signaling

The osmo-sensitive strain *yl-hog1Δ* could grow undisturbed on 0.75 M erythritol, while the same concentrations of glycerol and glucose were lethal (Figure 2). Moreover, the supplementation of erythritol in media with high concentrations of other tested carbon sources partially reinstated the growth of *yl-hog1Δ* (Figure 3 and Figure 5). The replacement of erythritol with other potential osmolytes such as mannitol or L-proline did not have a similar effect (Figure 3). These results indicate that erythritol plays a distinct role in the *Y. lipolytica* hyperosmotic stress response. The expected mechanism of protection was that erythritol is transported and accumulated inside the cell, to restore the osmotic balance. The further deletions of *EUF1* and *EYD1* seemed to confirm this possibility.

Eyd1 is one of the first enzymes of the erythritol utilization pathway [14,16]; a lack of it prevents incorporation of erythritol into metabolic pathways of the cell and is lethal when this polyol is the only carbon source (Figure 3). However, when erythritol was supplemented with other carbon sources, the strain *eyd1Δ yl-hog1Δ* could withstand the concentration of substrates that was lethal to *yl-hog1Δ* (Figure 5). This improvement of growth could be explained by inability to use the erythritol in the cell in any other way than accumulation as an osmolyte.

On the other hand, deletion of *EUF1*, a gene also important for erythritol utilization, had the opposite effect. The strain *euf1Δ yl-hog1Δ* could not benefit from the addition of erythritol to improve growth under osmotic stress (Figure 5). These results, although contradictory at first, may be of key importance in further investigating the role of erythritol in metabolism. Euf1 is a relatively recently discovered transcription factor [9], and for now it is known to regulate the transcription of only four genes—*EYD1*, *EYK1*, *EYI1*, and *EYI2* [16]—all of them being encoding enzymes of erythritol utilization and located in the cluster. If the only role of Euf1 was the regulation of this cluster, the deletion of *EUF1* and *EYD1* should have the same result. The observed difference indicates that Euf1 also targets other genes involved in erythritol metabolism.

So far, we have discussed the importance of accumulation of erythritol inside the cell. However, the most distinctive feature of the hyperosmotic stress response in *Y. lipolytica* is secretion of erythritol [3,35]. There is a possibility that erythritol simply leaks from the cell and constant production is necessary in order to maintain a sufficient concentration inside the cell. However, the observation that *Y. lipolytica* does not require activation of HOG in order to survive in a high erythritol concentration suggests that the secretion of erythritol might not be accidental leakage, but a component of a more complicated stress response mechanism. It should also be noted that the secretion is not a waste of a carbon source, as erythritol is ultimately re-assimilated [9].

That leads to the most important question: What mechanism makes erythritol harmless to the *hog1Δ* strain? Erythritol media have the same osmolality as equimolar glucose or glycerol media, so it should generate similar stress. The strong growth of *yl-hog1Δ* even at high erythritol concentrations indicates that *Y. lipolytica* must have a system to deal with osmotic pressure generated by erythritol, which is at least partially independent of Hog1. Poor growth of the strain *euf1Δ yl-hog1Δ* on plate cultures (Figure 5) and its inability to adapt during shake-flask cultures (Figure 6c) suggest that *EUF1* might be crucial for the possible HOG-independent stress response. For now, there are only a few proteins known to affect erythritol metabolism, but further studies on *EUF1* might help with the identification of more elements.

The first hypothesis is an erythritol-specific transporter or membrane channel. If Euf1 activates the transcription of genes responsible for erythritol membrane transport, *EUF1* deletion would disable the uptake of erythritol from the environment and in consequence also its accumulation. However, it might not be sufficient to explain the phenotype of *euf1Δ yl-hog1Δ*. The *euf1Δ* strains could still grow weakly on erythritol; thus at least part of the transport is independent of Euf1 [9].

The very strong growth of *yl-hog1Δ* on erythritol as a single carbon source suggests that when osmotic pressure is generated by erythritol, the activation of HOG is actually unnecessary. Thus, presence of erythritol might affect the activity of the HOG pathway. The idea that some Euf1-regulated proteins could be involved in cross-talk between cell signaling pathways was the motivation for creating the *EUF1 yl-hog1Δ* strain with *EUF1* overexpression.

In the presence of erythritol, *EUF1* expression is significantly increased [9]. Additionally, *EUF1* overexpression triggers the transcription of erythritol utilization proteins, even when erythritol is not present in the medium [16]. Therefore, we wanted to evaluate whether there is a possibility to obtain any benefits of erythritol supplementation by overexpressing *EUF1*.

The growth of the strain *EUF1 yl-hog1Δ* on plates did not differ from *yl-hog1Δ*, either in the presence or absence of erythritol; however, the differences become apparent in shake-flask cultures (Figure 6e). In the absence of erythritol, the strain did not develop faster, and adaptation was even slower than in the strain *yl-hog1Δ.* The reason for the weaker growth of *EUF1 yl-hog1Δ* might be overexpression of the whole erythritol utilization cluster, induced by overexpressed Euf1. During the first 24 h of culture a small amount of erythritol was produced and detected in the medium. Excessive activity of erythritol utilization enzymes might consume intracellular erythritol so fast that it could not be accumulated to work as an osmolyte.

On the other hand, when erythritol was abundant in the liquid medium, *EUF1 yl-hog1Δ* surpassed all other strains in terms of biomass increase and the glycerol utilization ratio was comparable to AJDU. This beneficial effect of EUF overexpression was not observed on agar plates supplemented with erythritol, which might be due to the inferior availability of erythritol from solid media. Thus, contrary to expectations, whatever mechanisms might be induced by Euf1, they only work when at least a little erythritol is present in the environment.

### 4.2. Auxiliary Role to HOG

The results of this study might indicate that erythritol is more important than glycerol for maintaining the osmotic balance in *Y. lipolytica*. Glycerol was even used as one of the stressing factors. However, it should be remembered that the experiments were conducted on *yl-hog1Δ* strains in which the main hyperosmotic stress response was disrupted. So far, we do not know what elements in the cell are affected by phosphorylated yl-Hog1 in *Y. lipolytica*. Yet, comparing the mechanisms known from other yeasts [18,19], there is a high possibility that the HOG-dependent response is also based on glycerol production and accumulation.

There is also the puzzling observation that erythritol supplementation was much more effective when the main carbon source was glycerol rather than glucose (Figure 4). This might be a result of similar chemical structure of erythritol and glycerol and thus the possibility to use the same elements of the cells, for example membrane transporter. Their higher expression on a glycerol medium may therefore contribute to a more effective use of erythritol supplementation. On the other hand, those two polyols might be both important for *Y. lipolytica* osmotic balance and have roles that complement each other, as was observed in other yeasts. In studies on *Moniliella megachiliensis*, glycerol production was observed as a rapid response to osmotic stress, while erythritol was more associated with oxidative stress and the long-term osmotic stress response [36,37]. The yeast *Trichosporonoides (Moniliella) oedocephalis* secretes glycerol and erythritol simultaneously. The deletion of its *HOG1* homologue caused a decrease in glycerol and a rise in erythritol production [38]. In the case of *Y. lipolytica* there are no reports about glycerol secretion under hyperosmotic conditions, but rises of intracellular concentrations of both glycerol [31] and erythritol were reported [23]. Thus, even among the erythritol-producing yeasts, the roles of glycerol and erythritol in osmoprotection are varied.

HOG is undoubtedly the pathway that regulates the main and most important response to osmotic stress. It should be noted that single modifications targeting erythritol utilization genes do not influence their sensitivity to hyperosmotic environment (Figure 4) [9,14,15,16], and only the combination with *yl-hog1Δ* revealed their role in stress adaptation.

Moreover, the supplementation of erythritol did not have any influence on growth (Figure 2), substrate utilization, or polyol production (Figure 6a) of the control AJDU strain. It indicates that the osmotic response activated by the functional HOG pathway in *Y. lipolytica* is so effective that external support is not necessary.

## 5. Conclusions

Studies on *Y. lipolytica* show that it shares some universal features of the hyperosmotic stress response that are conservative among yeast species, such as the central role of HOG signaling. However, the secretion and utilization of erythritol indicate the existence of other mechanisms, complementing the response generated by the HOG pathway. It might be less common, but probably not completely unique to *Y. lipolytica*, as erythritol is a carbon source or osmolyte for a group of other yeast species. Therefore, further research on the metabolism of this polyol might very important for full understanding of their osmotic stress response.

## Figures and Tables

**Table 1:** Strains used in the study.

**Strain**	**Genotype or Plasmid**	
***E. coli***		
*DH5α*		Laboratory strain
*DH5α*	pUC-Ura	[9]
*DH5α*	pUC-Ura-pF01650	This study
*DH5α*	pUC-Ura-ΔF01650	This study
*DH5α*	pQE80-ptHog1+Ura	[20]
*DH5α*	pUC-ura-ΔF01562	[9]
*DH5α*	pAD	[24]
*DH5α*	pAD-F01562	[9]
*DH5α*	pUB4-Cre1(JME547)	[25]
***Y. lipolytica***		
AJD	*MATA*, ura3-302	[26]
AJDU	*MATA*, ura3-302::*URA3*	This study
AJD *yl-hog1Δ*	*MATA*, ura3-302, *hog1Δ::URA3*	This study
AJD *euf1Δ*	*MATA*, ura3-302, *euf1Δ::URA3*	This study
AJD *euf1Δ* ura-	*MATA*, ura3-302, *euf1Δ*	This study
AJD *eyd1Δ*	*MATA*, ura3-302, *eyd1Δ::URA3*	This study
AJD *eyd1Δ* ura-	*MATA*, ura3-302, *eyd1Δ*	This study
AJD EUF1	*MATA*, ura3-302, pAD-*EUF1::URA3*	This study
AJD EUF1 ura-	*MATA*, ura3-302, pAD-*EUF1*	This study
AJD *euf1Δ yl-hog1Δ*	*MATA*, ura3-302, *euf1Δ*, *yl-hog1Δ::URA3*	This study
AJD *eyd1Δ yl-hog1Δ*	*MATA*, ura3-302, *eyd1Δ*, *yl-hog1Δ::URA3*	This study
AJD EUF1 *yl-hog1Δ*	*MATA*, ura3-302, pAD-*EUF1*, *yl-hog1Δ::URA3*	This study

## Supplementary Materials

The following are available online at https://www.mdpi.com/2073-4425/11/12/1424/s1, Figure S1: Growth of *Y. lipolytica* AJDU and *yl-hog1Δ* after 48 h of incubation on YNB agar plates with 0.3 M carbon sources.
