# Supplementary material for: HOG-Independent Osmoprotection by Erythritol in Yeast Yarrowia lipolytica
Source: Genes (Basel). 2020 Nov 27;11(12):1424. doi: 10.3390/genes11121424 (PMC7761004; doi:10.3390/genes11121424)
Supplement: Supplementary file 1 [file genes-11-01424-s001.pdf]

genes

Supplementary Material

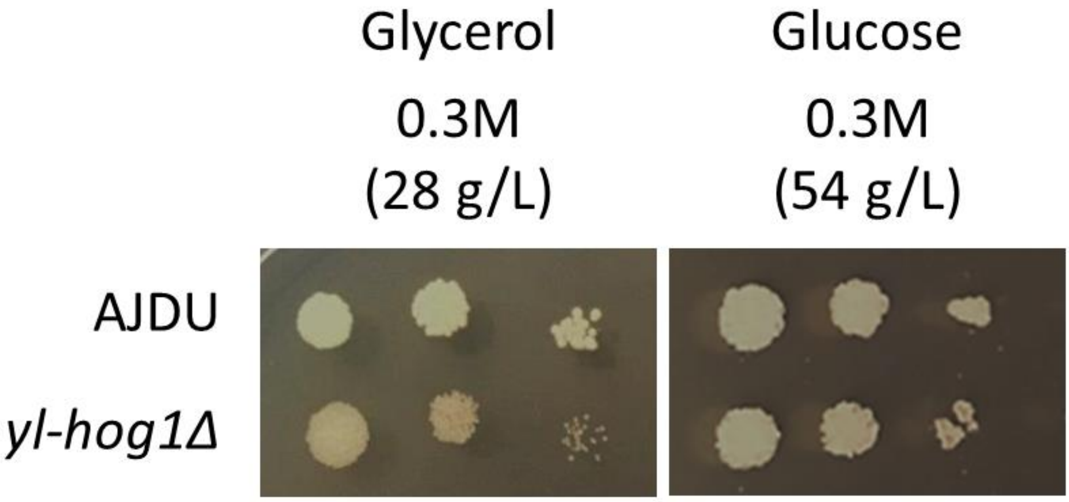

Figure S1. Growth of *Y. lipolytica* AJDU and yl-hog1Δ after 48 h of incubation on YNB agar plates with 0.3 M carbon sources.
